# Assessments of global drivers of vaccine hesitancy in 2014—Looking beyond safety concerns

**DOI:** 10.1371/journal.pone.0172310

**Published:** 2017-03-01

**Authors:** Melanie Marti, Monica de Cola, Noni E. MacDonald, Laure Dumolard, Philippe Duclos

**Affiliations:** 1 Department of Immunization, Vaccines and Biologicals, World Health Organization, Geneva, Switzerland; 2 Department of Paediatrics, Dalhousie University, IWK Health Centre and Canadian Center for Vaccinology, Halifax, Canada; Public Health England, UNITED KINGDOM

## Abstract

Vaccine hesitancy has become the focus of growing attention and concern globally despite overwhelming evidence of the value of vaccines in preventing disease and saving the lives of millions of individuals every year.

Measuring vaccine hesitancy and its determinants worldwide is important in order to understand the scope of the problem and for the development of evidence-based targeted strategies to reduce hesitancy.

Two indicators to assess vaccine hesitancy were developed to capture its nature and scope at the national and subnational level to collect data in 2014: 1) The top 3 reasons for not accepting vaccines according to the national schedule in the past year and whether the response was opinion- or assessment-based and 2) Whether an assessment (or measurement) of the level of confidence in vaccination had taken place at national or subnational level in the previous 5 years.

The most frequently cited reasons for vaccine hesitancy globally related to (1) the risk-benefit of vaccines, (2) knowledge and awareness issues, (3) religious, cultural, gender or socio-economic factors. Major issues were fear of side effects, distrust in vaccination and lack of information on immunization or immunization services. The analysis revealed that 29% of all countries had done an assessment of the level of confidence in their country, suggesting that vaccine confidence was an issue of importance.

Monitoring vaccine hesitancy is critical because of its influence on the success of immunization programs. To our knowledge, the proposed indicators provide the first global snapshot of reasons driving vaccine hesitancy and depicting its widespread nature, as well as the extent of assessments conducted by countries.

## Introduction

Vaccine hesitancy is defined as a delay in acceptance or refusal of vaccines despite available vaccination services. It is a complex, context-specific, and rapidly changing global phenomenon that varies across time, place and vaccines. [1]

From 2002–2005, a pilot question was included in the annual World Health Organization (WHO) and United Nations Children’s Fund (UNICEF) Joint Reporting Form (JRF) in order to monitor and understand the increasing number of reports on vaccine hesitancy. The JRF was sent to national immunization managers globally asking whether they had responded to negative media coverage about vaccines within the previous year. Data showed that negative media coverage was reported by countries in all WHO regions. This survey question pointed to the extent of vaccine hesitancy already emerging even over a decade ago. [2]

The recent Decade of Vaccines (DoV) Global Vaccine Action Plan (GVAP) [3] 2011–2020 set 6 Strategic Objectives for the decade, with proposed indicators to monitor and evaluate progress. The Strategic Advisory Group of Experts (SAGE) on Immunization Working Group on Vaccine Hesitancy developed 2 new indicators for the second Strategic Objective: Individuals and communities understand the value of vaccines and demand immunization as both their right and responsibility. These indicators were as follows: 1) The top 3 reasons for not accepting vaccines according to the national schedule in the past year and whether the response was opinion or assessment-based 2) Whether an assessment (or measurement) of the level of confidence in vaccination had taken place at national or subnational level in the previous 5 years. [4][5] The global launch of these indicators took place in the 2015 JRF to collect country data for 2014 (referred to as 2014 JRF data).

The objective of including the indicators in the JRF was to allow for identification of the reasons driving vaccine hesitancy globally and evaluate which countries have conducted an assessment of the level of confidence. The initial inclusion of these indicators was shown to be successful for determining the 3 main drivers of vaccine hesitancy in 2014 and establishing the percentage of countries that have conducted an assessment. These indicators also facilitate the monitoring of changes and progress over time.

## Methods

In an effort to monitor the existence, global progress, and related determinants of vaccine hesitancy WHO and UNICEF included the vaccine hesitancy indicators in the JRF with data collection for 2014. [6]

The JRF is a standardized questionnaire that is sent to all 194 WHO/UNICEF Member States on an annual basis. The JRF was developed as a tool to collect data on immunization coverage, reported cases of vaccine-preventable diseases, immunization schedules and on indicators related to immunization program performance such as the establishment of national technical immunization advisory groups. [7] Most often, it is national immunization managers who complete the questionnaire.

The JRF was sent to countries in the first quarter of 2015; hence the collected data represents the country situation in 2014.

**Fig 1:**
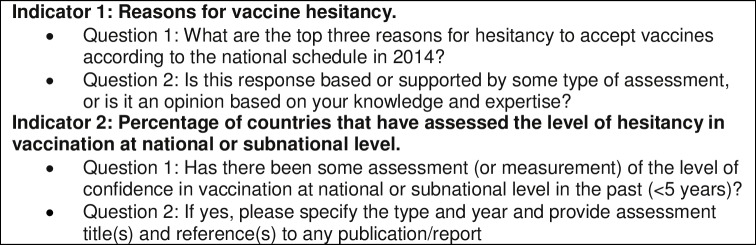
Indicators of vaccine hesitancy included in the 2014 JRF.

In the JRF, there were 2 indicators related to vaccine hesitancy each represented by 2 questions. Question 1 of Indicator 1 was structured as an open-ended question inquiring about the top 3 reasons for not accepting vaccines in the last year. Immunization managers were given a free text field within a spreadsheet to provide 3 responses. The questionnaire was designed for 3 reasons or less. The second indicator was phrased as a closed dichotomous question on whether an assessment of vaccine hesitancy within the previous 5 years (yes/no), followed by a free text field to provide title and reference of the respective assessment (Fig 1).

The JRF question response rate was calculated by region. For analysis, all WHO/UNICEF Member States were included as the denominator. Data were compiled using Microsoft Excel 2010. Data were analysed using Stata version 12.0 (StataCorp. 2011. Stata Statistical Software: Release 12. College Station, TX: StataCorp LP.).

The results were stratified by WHO region, [8] development status according to the 2015 World Bank national income status categories [9] and the country’s population size using the United Nations (UN) population estimates. [10]

**Fig 2:**
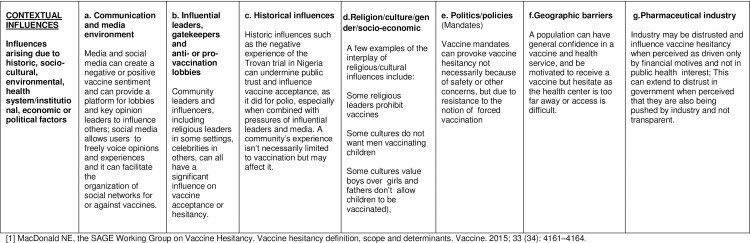
Matrix of determinants of vaccine hesitancy. Contextual influences.

**Fig 3:**
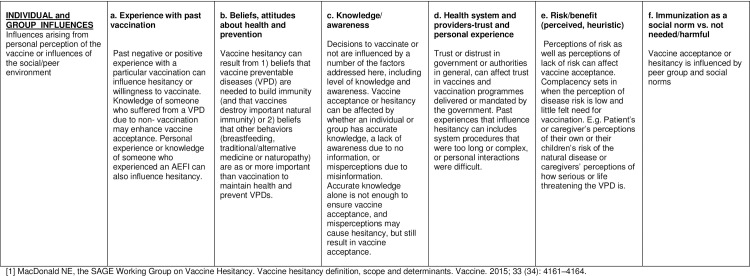
Matrix of determinants of vaccine hesitancy. Individual and group influences.

**Fig 4:**
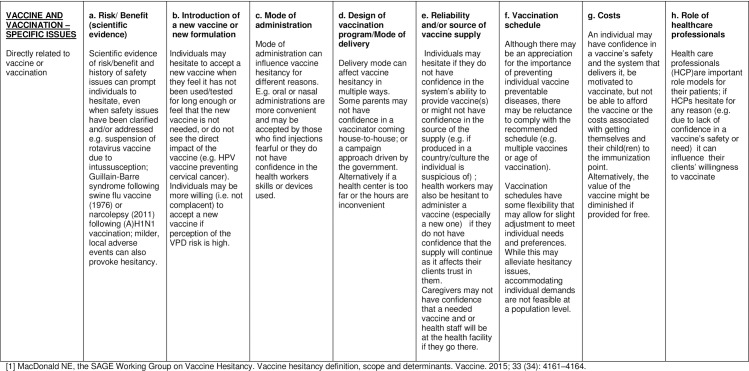
Matrix of determinants of vaccine hesitancy. Vaccine and vaccination-specific issues.

The main factors influencing vaccine hesitancy in each country in 2014, were analysed using both quantitative and qualitative methods. As a means of interpretation, the responses were categorized according to the matrix of determinants developed by the SAGE Working Group on Vaccine Hesitancy (Figs 2–4). [1] The matrix of determinants displays the specific drivers influencing the behavioural decision to accept, delay or reject some or all vaccines in 3 different categories: contextual influences, individual and group influences, and vaccine and vaccination-specific influences.

Categorization was performed individually by 2 researchers. If the researchers came to different conclusions, a content-expert was consulted to make an executive classification.

The results were summarized according to the frequency with which the determinants were cited. Furthermore, similarities and differences in related codes across distinct original sources/contexts are discussed.

The analysis focused on low- and lower-middle-income countries with the largest population size, which are the priority countries for WHO.

If an assessment title was provided for Indicator 2, it was assumed that an assessment had taken place even if there was no response to Question 1 of Indicator 2. Assessments were grouped by target population and related vaccine for available qualitative data.

## Results

### Response rate

Out of all 194 WHO Member States who were requested to fill out the 2014 JRF, 185 (95%) returned the completed form and 154 Member States (79%) provided any information for either of the 2 vaccine hesitancy indicators. The response rate of countries by region ranged from a high of 100% in the South East Asian Region (SEAR), 89% in the Region of the Americas (AMR), 87% in the African Region (AFR), 76% in the Eastern Mediterranean Region (EMR), 70% in the European Region (EUR) to a low of 67% in the Western Pacific Region (WPR).

### Reasons for vaccine hesitancy

Of 194 Member States, 141 (73%) provided at least one reason for vaccine hesitancy (Table 1).

**Table 1:** Number and percentage of countries that responded to the question on the top three reasons for vaccine hesitancy in 2014.

	ALL REGIONSn (%)	AFR n (%)	AMR n (%)	EMR n (%)	EUR n (%)	SEAR n (%)	WPR n (%)
Member States providing at least one reason	141 (73%)	34 (72%)	27 (77%)	15 (71%)	36 (70%)	11 (100%)	18 (67%)
Member States providing no reason	53 (27%)	13 (28%)	8 (23%)	6 (29%)	17 (39%)	0 (0%)	9 (33%)
Total Member States	194	47	35	21	53	11	27

**Fig 5:**
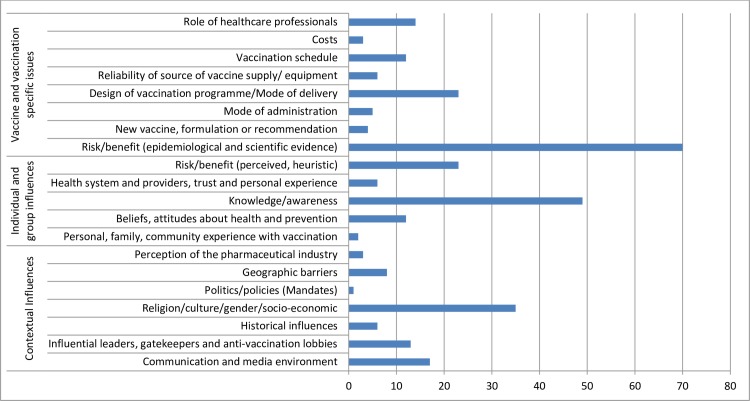
Frequency of main themes indicated as top three reasons for vaccine hesitancy within all WHO regions.

Forty-nine (25%) Member States provided the requested 3 reasons, 25 (13%) Member States provided 2 reasons and 141 (73%) countries provided at least 1 reason for vaccine hesitancy in the previous year. The data collected for vaccine hesitancy were mapped against the matrix of determinants of vaccine hesitancy and grouped according to the 3 categories of specific influences: contextual, individual and group, as well as vaccine and vaccination (Fig 5).

Overall, the matrix of determinants was able to capture the diverse reasons provided by the immunization managers besides a small subset that were not classifiable (n = 14; e.g. “trust”, “no confidence” or “did not want to be vaccinate”). Concerns related to the risk and benefit of vaccines were the most commonly cited reasons leading to vaccine hesitancy. Safety issues including fear of side effects and safety of the vaccine were grouped within this category and were especially prominent in EUR, SEAR and WPR. Issues around religion, culture, gender or socio-economic status, particularly in AMR were common. However, the religious sect was usually not specified. When an explanation was provided, the reasons ranged from Rastafarian belief, interference to spiritual development, no halal certification of vaccines, the disease is considered a divine will to anthroposophical beliefs.

The most frequently cited determinant of vaccine hesitancy by immunization managers from AFR and EMR was knowledge and awareness issues such as a lack of knowledge or information on vaccines and their benefits as well as low awareness of the need for immunization.

**Fig 6:**
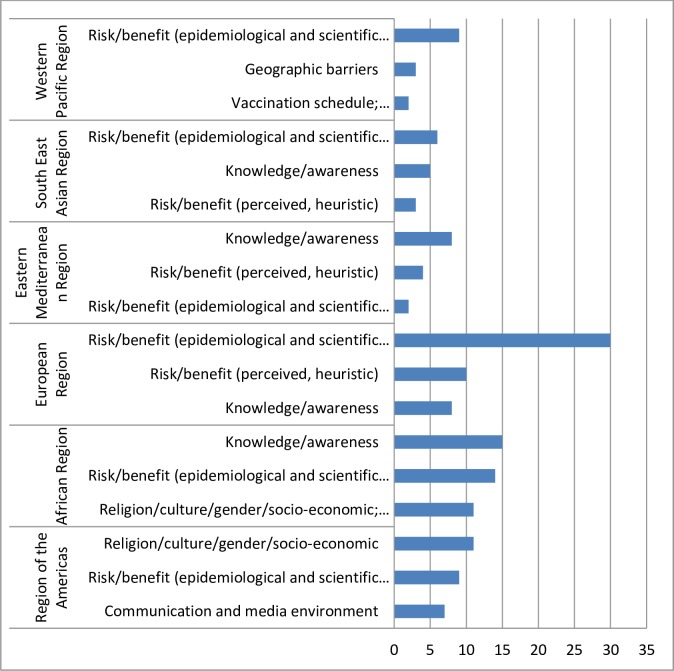
Top three reasons for vaccine hesitancy by region.

The top 3 reasons in each region are summarized in Fig 6. When comparing the results across regions, there was consistency in the causes provided across regions. However in AMR, communication and the media environment were often cited. Some immunization managers specifically pointed to the impact of increasing anti-vaccination lobbyists and misinformation quoted in the media as a driving factor for vaccine hesitancy. Two countries reported Ebola as a major barrier to vaccination in 2014. For some EMR and AFR countries, the design of the vaccination program was highlighted such as service-provider related issues including inconvenient times and opening hours for vaccination clinics, caregivers forgetting their appointments as well as the repetitiveness of immunization campaigns and preference for private vaccinators. A factor frequently cited in EUR was the low perceived risk of vaccine preventable diseases (complacency) and the lack of seeing the benefit of immunization, i.e. negligence and missing motivation to receive the recommended vaccines.

**Fig 7:**
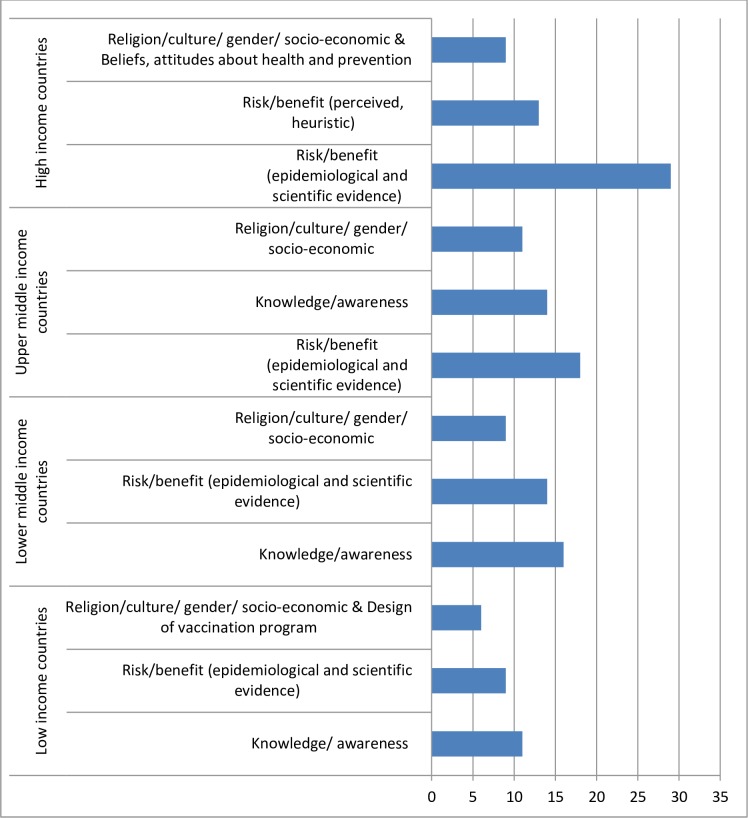
Top three reasons of vaccine hesitancy by level of income globally.

Analysis of the drivers of vaccine hesitancy by income status indicated that in low- and lower-middle-income countries a lack of knowledge and awareness of the required vaccines, immunization or immunization services were the most frequently cited contributors to vaccine hesitancy. In upper-middle-income countries, the reasons evolved around risk/benefit (epidemiological and scientific evidence) of immunization, in particular issues related to adverse events following immunization (AEFIs) and safety of the vaccine. In high-income countries, the main reason reported by immunization managers pertained to vaccine safety issues classified within the determinant of “Risk/benefit (epidemiological and scientific evidence)” (Fig 7).

Vaccine hesitancy issues related to religious beliefs (”Religion/culture/gender/socio-economic”-determinant) were prominent across all strata of income and WHO regions. In AFR, the predominant reasons in low- as well as in upper-middle-income countries and high-income countries were related to knowledge and awareness issues. In low-income countries in EMR, vaccine hesitancy was most often associated with geographic barriers.

In assessing whether these responses were evidence-based or opinion-based, relying on the expertise of the immunization manager in the 141 countries who responded with at least 1 reason, it was opinion-based for 80 countries (57%), evidence-based for 48 (34%) on evidence, and 13 (9%) did not specify.

Of the 10 world’s largest countries by population size according to 2015 UN estimates (China, India, United States of America, Indonesia, Brazil, Pakistan, Nigeria, Bangladesh, Russia and Japan), 5 are classified as low-middle-income countries (the remaining 5 as upper-middle or high-income countries). In the 5 low-middle-income countries, 4 countries provided a total of 11 reasons for vaccine hesitancy in their country. The most frequently cited reasons for hesitancy were lack of awareness and information gaps, classified within the knowledge/awareness determinant (Knowledge/awareness n = 5), followed by fear of adverse events (Risk/benefit (epidemiological and scientific evidence) n = 3). Other reasons cited, each named once, related to the low perception of risk posed by the disease, belief in use of local remedies and rumours. Three of the 4 countries indicated that an assessment had been done and provided specific information on the assessment.

Globally, only 7% of the immunization managers (n = 13) indicated no knowledge of vaccine hesitancy within their country, noted that vaccines were well received by the population, or both. Of these countries, 4 were in EUR (8% of the region’s Member States), 4 were in EMR (19% of the region’s Member States), 3 were in AMR (9% of the region’s Member States), and 2 were countries in SEAR (18% of the region’s Member States). According to the World Bank income level classification, 3 countries were low-income countries, 6 lower-middle-income, 2 upper-middle-income and 2 high-income countries.

### Assessment of vaccine hesitancy; confidence component

An assessment of vaccine confidence had been done within the previous 5 years in 56 (29%) Member States; 64 (33%) reported no assessment and 74 (38%) did not provide a response.

Fifty-three (percentage) countries provided detailed information or assessment titles and references to any publication or report on vaccine hesitancy (S1 Table). Five countries reported on multiple surveys and 1 country indicated an annual survey. The number of reported assessments also increased over time in the surveyed time period. The highest number of reported assessments were indicated to have taken place in 2014 (n = 19), compared to 10 in 2013, 9 in 2012, 3 in 2011 and 4 in 2010. Assessments were conducted in all regions in 2014.Countries in AFR conducted the most assessments: 2 in 2010 and 2011, 6 in 2012 and 5 in 2013 and 2014. Countries reported having conducted an assessment in 45% of SEAR countries (n = 5/11), 28% of EUR countries (n = 15/53), 24% of EMR countries (n = 5/21), and 22% of WPR countries (n = 6/27).The type of assessment used, when provided, was as follows: immunization coverage surveys (review of the Expanded Program on Immunization) (n = 11) followed by knowledge, attitude, behaviour and practices studies (n = 10) and post-campaign surveys (n = 2). Other examples listed were drop-out survey, online survey, formative research, polling and demographic and health survey (n = 1).

Confidence issues also varied depending on the type of vaccine. The most frequently named were routine childhood vaccines (unspecified) (n = 20), measles vaccine (n = 3), HPV and seasonal influenza (n = 2) as well as new vaccines and tetanus vaccine (n = 1).

When indicated or retrievable, target population of the assessments were most commonly parents and caregivers (n = 7), adults (n = 1), young women (n = 1) or health care workers (n = 1). Distinguishing whether the assessment was conducted at the national or subnational level was not feasible in the majority of cases based on the answers provided.

## Discussion

No indicators have been identified to date that will facilitate the measurement of various vaccine hesitancy determinants accurately across all contexts. [2] Given the complexity and length of the JRF adding, or changing a quesiton or both has to be done with care and caution. As such, the new data generated must be compelling enough to justify the change.

The results of the vaccine hesitancy indicators from the 2014 JRF data provide useful insights for global program planning and for tracking of hesitancy. However, they still fall short of measuring the depth and breadth of the hesitancy problem. Nevertheless, the indicators support tracking of perceived reasons for vaccine hesitancy within a country and summarize the kind of surveys conducted thus far that have aimed to assess vaccine confidence.

The findings from the 2014 JRF data survey are also consistent with previous observations that vaccine hesitancy varies by time, place, and vaccine. In Ebola affected countries, [11] people were afraid to access health care centres due to the potential risk of Ebola transmission, an example of a temporal determinant. In EUR, the fact that vaccine preventable diseases were not perceived as a risk illustrates complacency with immunization as defined by the matrix of determinants. This is especially important because this region has been struggling with re-emergence of some vaccine preventable diseases such as measles, mumps and pertussis in spite of well-established and reliable vaccination programs. In particular, the measles outbreaks in the region have been attributed to intentional under-vaccination. [12] As a result, interventions to address hesitancy in Ebola affected countries will be quite different than in countries where complacency is a key factor, reinforcing that vaccine hesitancy is highly context specific.

Furthermore, as observed in a previous study, [13] the WHO matrix of determinants was demonstrated to be robust, as only a few factors identified in the survey fell outside of the scope of the matrix.

The majority of reported reasons for vaccine hesitancy were related to vaccine and vaccination specific issues. Globally, the most frequently reported explanations were related to the risk/benefit determinant (epidemiological and scientific evidence). In particular, vaccine safety issues and fear of AEFIs were cited, which reinforces the need to address this concern globally and locally. However, since alleged vaccine safety issues are known to have triggered prominent episodes of vaccine hesitancy in developing as well as developed countries, [14] immunization managers may have felt compelled to place it on the list, Therefore, the information provided by the immunization manager may be subjective, which highlights one general limitation of this indicator. As a result, the information provided may not necessarily reflect the situation in the country or at the sub-national level and specific determinants may be over- or underreported.

The second most frequently cited reason for vaccination hesitancy was knowledge and awareness issues. This was a common concern in low-and lower-middle-income countries. Addressing the gaps in knowledge may help decrease vaccine hesitancy. However, determinants such as education enable and well as hinder immunization uptake. [15]

The third most common reason for vaccine hesitancy was religious and cultural issues, particularly religion. However, the religion or belief was often not specified. A review by Grabenstein et al. [16] of the populous faith traditions of the world including Hinduism, Buddhism, Jainism, Judaism, Christianity, and Islam found minimal evidence of “canonical bases for declining immunization”. This suggests that in countries where religion and culture are barriers to immunization, more work needs to be done with religious leaders to determine the precise concerns and then review if this truly has a religious basis or not. One must be aware that sometimes religious concerns are cited to cover up underlying concerns or to bring attention to a community where problems other than vaccines need to be addressed.

Limitations in this analysis approach were observed when categorizating Indicator 1 by the matrix of determinants. In some instances answers fit in more than one category. For example, “Complacency of well off families” could be grouped as perceptions of a lack of risk of vaccine-preventable diseases “Risk/benefit (perceived, heuristic)” as well as “Immunization as social norm” with vaccine hesitancy being influenced by the named peer group. Moreover, imprecision of the information provided demonstrated challenges for grouping. It was unclear whether “Fever” relates to fear of AEFIs, “Risk/benefit (epidemiological and scientific evidence),” or “Experience with past vaccination”. Furthermore, the classification of the provided reasons may be subject to personal perception. If no reason was provided, it was unclear whether the main 3 reasons were unknown to the immunization manager or whether no hesitancy was presumed to be present in the country. Evaluating vaccine hesitancy by immunization managers on a yearly basis may improve the quality of the answers or bring forth more concerns as immunization mangers become comfortable with the query.

With respect to the second indicator, the number of assessments on vaccine confidence might serve as a proxy for the number of countries being confronted or facing difficulties with vaccine hesitancy. However, the current JRF question focused more narrowly on “confidence” instead of broadly on hesitancy. This led to some difficulties in interpreting the results as some reports clearly were assessing hesitancy more broadly than others. Changing the first question of the second hesitancy indicator to a query about the conducted assessments on “vaccine hesitancy” rather than about assessments on “confidence in vaccination” may be advisable to allow the indicator to encompass the broader scope of vaccine hesitancy and avoid confusion among immunization managers. Nevertheless, the 2014 findings are of interest as they showed a broad range and breadth of studies being undertaken in this discipline across the globe. Furthermore, it is noteworthy that the number of assessments per year has increased over the time period surveyed. This increase may be related to recall bias, in particular as staff rotate, but also may reflect a true increase in concern in this area. Future JRF reviews will provide more evidence to show if this observation is a true upward trend.

Addressing vaccine hesitancy requires an understanding of the magnitude and setting of the problem as well as a diagnosis of the root causes using valid and reliable measures deployed in populations large enough to draw confident conclusions. [17] Vaccine hesitancy must always be viewed in the context of other factors contributing to un- or under-vaccination. The 2 indicators evaluated herein may assist with advocating and raising awareness of vaccine hesitancy within countries. They may also stimulate action, thorough assessment and identification of the specific determinants of vaccine hesitancy within a country or sub-country level. Evidence-based strategies can only be tailored to address these causes successfully if the underlying determinants of hesitancy in that context are well understood.

Overall, the results gathered from the 2 indicators highlight specific factors contributing to vaccine hesitancy across regions and suggest that vaccine hesitancy affects all countries regardless of income status. These indicators are of particular importance because to date there is no global assessment, to our knowledge, of the causes of vaccine hesitancy. Both indicators are useful in that they will not only raise awareness about hesitancy among countries and encourage the development of targeted strategies to reduce vaccine hesitancy, but also promote monitoring progress to address vaccine hesitancy across the regions and countries over time.

## Supporting information

S1 Table List of assessments by country as indicated in the 2014 JRF data. (DOCX)
