# Supplementary material for: Assessments of global drivers of vaccine hesitancy in 2014—Looking beyond safety concerns
Source: PLoS One. 2017 Mar 1;12(3):e0172310. doi: 10.1371/journal.pone.0172310 (PMC5332020; doi:10.1371/journal.pone.0172310)
Supplement: S1 Table — (DOCX) [file pone.0172310.s001.docx]

S 1 Table: List of assessments by country as indicated in the 2014 JRF data.

| Australia | The National Centre for Immunisation Research and Surveillance evaluation of the National Human Papillomavirus Vaccination program in Australia. Includes a review of barriers to vaccination. |
| --- | --- |
| Bangladesh | EPI Coverage Evaluation Survey 2014 |
| Benin | EDS4 2012 |
| Bolivia | ENCOVA |
| Botswana | Botswana Uppen partnership study on vaccine hesitancy study |
| Burkina Faso | Comprehensive Review of the ENP |
| Cameroon | Survey of Knowledge, Attitudes and Practices of populations for vaccination in Cameroon (CAP) |
| Central African Republic (the) | CAP Survey, external review of the EPI 2012 |
| Chad | Immunization coverage survey of children aged 12-23 months, children 0-11 months and women who gave birth in the last 12 months in the districts of N'djamena health region |
| Comoros (the) | ECV 2013: Survey of immunization coverage for measles post campaign and routine immunization in the Union of Comoros |
| Congo (the) | KAP survey 2013/ 2014 external review |
| Côte d'Ivoire | External review of the EPI in 2012; routine immunization coverage survey in 2013 and the coverage survey campaign for measles, 2014 |
| Croatia | Online interview conducted in 2014/2015 |
| Democratic Republic of the Congo (the) | Anthropological study (2014) and Survey Post TMN campaign |
| Denmark | Wójcik OP, Simonsen J, Mølbak K, Valentiner-Branth P. Validation of the 5-year tetanus, diphtheria, pertussis and polio booster vaccination in the Danish childhood vaccination database. Vaccine 2013;31(6):955-959. |
| Dominican Republic (the) | External Institution polling, Support PAHO - CDC (Gallup) |
| Ecuador | Characterization Study: Internal Mobilization Of The Population Of Pastaza , Cotopaxi, Tungurahua And Chimborazo And Its Relationship With Measles Outbreak |
| Egypt | 2014 monitor report |
| Estonia | Seasonal influenza vaccination attitude report found at: http://terviseamet.ee |
| Fiji | EPI Coverage survey Report 2013 |
| Gabon | EPI review in 2012 (report) |
| Georgia | Formative Research |
| Germany | Representative survey targeting parents of children aged 0-13 years in 2010 (German Federal Centre for Health Education); assessment report: http://www.bzga.de/forschung/studien-untersuchungen/studien/?sid=10&sub=64 |
| Ghana | KABP Study: Introduction of new vaccines into the routine EPI Services in Ghana – Knowledge, Attitude, Beliefs and Practice Study |
| Guinea | External review of the EPI in 2011; EDS 2012 |
| Indonesia | Drop-out Study in Java Provinces |
| Iran (Islamic Republic of) | Poorolajal J, Khazaei S, Kousehlou Z, Bathaei S, Zahiri A. Delayed vaccination and related predictors among infants. Iran J Public Health. 2012;41(10):65-71. |
| Ireland | National telephone survey 2013: adults (unpublished data) |
| Kyrgyzstan | Formative research perception of the quality of immunization , UNICEF 2013 |
| Lesotho | Lesotho coverage report Jul 2014 |
| Liberia | KAP Study |
| Mali | Immunization coverage survey 2010 |
| Micronesia (Federated States of) | Pohnpei assessment survey 2010 |
| Myanmar | Perceptions towards Access and Utilization of Routine Immunization Services |
| Netherlands (the) | Chapter 8 of thesis Irene Harmsen found at: http://digitalarchive.maastrichtuniversity.nl/fedora/get/guid:072c7383-8a0a-4d67-87cb-615c3217b5f5/ASSET1 |
| New Zealand | Immunisation Audience Research found at: http://www.health.govt.nz/system/files/documents/publications/immunisation-audience-research-1feb-2011-final.pdf |
| Niger (the) | KAP survey in September 2014 |
| Norway | Stefanoff P, Mamelund S, Robinson M, Netterlid E, Tuells J, Bergsaker MAR, et al. Tracking parental attitudes on vaccination across European countries: The Vaccine Safety, Attitudes, Training and Communication Project (VACSATC). Vaccine 2010;28(35):5731-5737. Found at: http://www.fhi.no/dokumenter/7cc1dcbdcc.pdf |
| Pakistan | KAPB Study 2014 |
| Peru | Evaluacion Intrenacional OPS |
| Republic of Moldova (the) | 2012 UNICEF |
| Russian Federation (the) | Annually through questionnaires during the ENI in several regions of the country |
| Senegal | Perception survey on new vaccines |
| Sierra Leone | EPI Coverage Survey 2013 |
| Somalia | Somaliland Immunization |
| Sudan (the) | Rapid assessment (Mini Survey) |
| Switzerland | Measles population survey 2012; HPV survey among young women 2014; influenza population survey 2014 |
| Tajikistan | Analytical Report |
| Thailand | The national high risk population for incomplete vaccination survey |
| Togo | CAP Study |
| United Kingdom of Great Britain and Northern Ireland (the) | Annual surveys of parents’ attitudes to childhood immunisation studied over a twenty year period. Ref. Dept of Health. TNS-BMRB Report |
| Viet Nam | Rapid assessment about trust in immunization for children under 12 months of age, among parents and health workers, UNICEF 2014, Unpublished. |
| Zambia | KAP Study (UNICEF) |
